# Nursery Government in Its Sanitary Aspects

**Published:** 1856-07

**Authors:** T. Herbert Barker


### ORIGINAL COMMUNICATIONS.

### NURSERY GOVERNMENT IN ITS SANITARY ASPECTS.

By T. HERBERT BARKER, M.D., F.R.C.S.

(*Continued from p.* 58.)

*Proper Diet of Infancy.* It might be supposed that every parent and every nurse would understand that the best nutriment for the babe is that supplied by the maternal breast; but medical interference is often required to enforce this simple truth, to explain its full importance, to show the difficulty of finding a good substitute for the natural diet, or to reprove the errors or unworthy motives that would allow any slight inconvenience to deprive a child of its proper nutrition. Unfortunately, it is a fact too frequent in this country, that the infant is consigned to the risk of artificial feeding, or to the care of a hired nurse, in cases where no necessity for such departure from nature's order exists. If mothers could see and understand this evil in all its bearings,—if they could follow the destiny of the child banished from the breast, and watch its gradual, daily decline, and death for want of its natural food—they would, we believe, in many cases, make greater efforts to fulfil their duty to their own offspring.

In cases where, happily, it is resolved that the child shall have its natural diet, some hours, or perhaps days, may elapse before the secretion of milk is ready. Here medical advice is required to correct the prejudices of nurses, who do not understand that, in ordinary circumstances, an infant is well able to bear a fast of twelve hours immediately after its birth. In this interval, while the flow of the mother's milk is deferred, the nurse is too commonly anxious to supply the supposed want by pouring gruel or panada into the child's stomach; and the consequent indigestion and flatulence are, perhaps, treated with "dill-water" or some other carminative. This process is useless and mischievous; but when there occurs a delay of some days in the secretion of milk, the child must be supplied with the nearest possible imitation of its natural diet. This will be found in a mixture of cow's milk and warm water in equal parts, with the addition of a small quantity of refined sugar.

In many instances, nurses are found too ready to suggest to the mother that her strength is inadequate to the duty of suckling her infant. The experienced medical man will easily put aside imaginary difficulties. He will assure the mother that her power to nourish the infant is sufficient, without the aid of artificial food. The child will be rescued from false treatment, and restored to the breast; and the secretion of milk, thus encouraged by its natural stimulus, will soon be found copious enough.

The diet of the nursing mother should be the same in quality as before her confinement; but, through increase of appetite, she may require a somewhat larger quantity of food, which should always be simple—not rich and stimulating. The common error of partaking too freely of wine, ale, or porter during the period of suckling, should be avoided. Occasionally the action of the bowels may require the aid of medicine; but aperients should be selected carefully, as they must affect the quality of the milk, and consequently the health of the child. Frequent gentle exercise in the open air, the use of a tepid shower-bath, or sponging the body with tepid or cold water, may be strongly recommended to the nursing mother.

So far, we have proceeded on the supposition that the mother obeys the dictates of nature and nurses her own child. It would be impossible to insist too strenuously on this point, as it seriously affects the question of public health. Let not the infant, for any trivial reasons, be exiled from the mother's breast. Let nothing less than disease, or physical inability, deprive the child of its natural nutrition.

In cases where the mother either cannot, or, on medical authority, must not suckle her own child, the choice of a suitable wet-nurse will require consideration. If we are careful of the quality of animal food given to adults, how much more studious ought we to be in selecting the first nutriment of the sensitive infant! In age, constitution, and temperament, it is well that the wet-nurse should, as nearly as possible, resemble the mother: of course, with the exception of any morbid traits in the latter. Sound health, regular appetite, clear complexion, good temper, freedom from every sign of cutaneous or scrofulous diseases—these are some of the chief marks of the eligible wet-nurse. Her milk should be thin, and of a bluish-white colour and sweet taste; and, after standing awhile in a vessel, should throw up a considerable quantity of cream. If dropped in water, it should assume a light cloudy appearance, and not fall to the bottom in thick drops. The diet of the wet-nurse should be generous, yet temperate. Indulgence in ardent spirits must be strictly prohibited; and, if stimulus is required, a moderate quantity of pure malt liquor, such as Bass's or Allsopp's ale, may be allowed. When the nurse is—as she should be—competent to the discharge of her duty in nourishing the infant, no other kind of food should be given. To give solid food, such as *bouillie* (flour boiled in cow's milk), while there is a good supply of far superior nutriment in the breast, is a gross folly.

Mauriceau has recorded the case of a healthy child which was fed, on the third day after its birth, with *bouillie* (or flour boiled in milk). The consequence was, that the infant died under a severe attack of colic attended with convulsions. There can be no need of any artificial food, if, during the first five or six months, the infant is applied to the breast at regular intervals of about three or four hours, by night as well as by day. The appetite of the child can be thus duly understood and satisfied by regularity of attention. "A single ounce of milk", says Dr. Combe, "well digested, will nourish more than double the quantity when it oppresses the still feeble stomach."

*Diet of a Premature Child.* Perhaps this will be the most suitable place for a few hints on the nursing of children prematurely born. Immediately on the premature birth of an infant (when there is hope that it may live), a healthy nurse should be found, so that the infant may at once be nourished with natural food, instead of waiting for the appearance of the mother's milk. It is desirable, in such a case, that the nurse should have small nipples, such as may be inclosed within the lips of the infant. If it be too feeble to suck, the artificial milk, before alluded to, must be administered by means of a small teaspoon. Its feeble stomach and digestive powers will require but a small quantity at a time, perhaps not more than two or three teaspoonfuls of the mixture of milk and water, but at shorter intervals—say every hour or two, unless longer sleep should prevent.

There is a popular prejudice in favour of children born at the seventh month of pregnancy, and against the surviving of eight months' children. This notion, however, is entirely without reasonable foundation ; for it is a fact ascertained beyond doubt, that the seven months' child has a less chance of living than one of eight months, as the latter has a less chance than the child at the full period : but, with careful and delicate attention, both the seven months' and the eight months' child will be likely to live. It need hardly be said, that attention to a strictly natural diet is of especial importance in such cases.

*Weaning.* Weaning implies a *gradual* withdrawal of the infant from the breast, and a careful substitution of other food in the place of the nurse's milk. The younger the infant, the greater the care required in the choice of food adapted to its digestive organs. We are easily guided by nature to determine, in all normal cases, the proper time of weaning. It is clearly indicated by the appearance of teeth; and, therefore, should generally take place between the seventh and the twelfth month. Here we would offer a word of caution against protracted suckling. Constitutions capable of nursing with impunity a strong healthy child for some length of time beyond twelve months, are but rarely met with; and medical men frequently observe the injurious effects of such attempts, both to mother and child. What these injurious effects are, it is not our province in this chapter to describe, but the opportunity could not be allowed to pass without thus briefly adverting to them.

If weaning have been determined upon, a proper quantity of the best diet should now be regularly given at intervals of about three hours each, and the child should no longer be disturbed by a want of food during the night. To avoid this, a sufficient meal should be given a short time before the infant goes to rest, and it should be fed early every morning. A common plan is to wean the child during the day, and for some time to continue to nurse it during the night; and this is unobjectionable, provided the mother do not allow the child to convert the night season into a regular period of feasting. Should the child fall into this bad habit, the mother will rise in the morning exhausted and unrefreshed, and the child's digestive organs will probably become disordered. To guard against this, let the breast be given at longer intervals during the night, and its artificial food as late at night and as early in the morning as convenient.

But of what materials should the diet now consist?

Food during and after weaning should not differ too much from the qualities of the previous milk diet. To make the change from the breast to the new diet gradual, the infant should be allowed to take a little soft and mild food, such as wholesome bread steeped in milk and water, as soon as any teeth have appeared, and thus he will be gradually prepared to leave the breast. But, even after weaning, the diet should still be mild, such as rice, oatmeal, gruel, panada, or biscuits steeped in water, with the addition of a little milk and sugar, and gradually other similar articles of food may be added. The yolks of lightly boiled eggs are rich in nutritious matter,

The *lait de poule,* a French preparation, may be made by shaking or beating up the yolk of an egg in more than half a pint of water sweetened with a little sugar. Hard's farinaceous food, Leman's tops and bottoms, or Dodson's biscuit-powder, may be tried ; or, if these disagree with the stomach, weak beef-tea, veal or mutton broth, without fat, and mixed with an equal quantity of farinaceous food and a few grains of salt. Bullock's Semola, a preparation of the gluten from wheat, is a light, digestible, and nutritious article of food for infants and invalids, which I have frequently recommended, and found to agree well with the stomach, when other preparations and kinds of food had failed. No general rule as to the particular food which will be suitable during the process of weaning can be laid down ; for in practice it is repeatedly observed that one kind of food, which agrees remarkably well with one child, as decidedly disagrees with another. The general principles which have been insisted on must be followed, namely, of giving sufficiently thin and light food, and not in too large quantity ; and selection must be made, after sufficient trial, of that particular preparation which suits best the stomach of the child.

Salt and sugar are the only proper condiments to be allowed in the food of children. Though I have strong objections to the use of sweetmeats, I would not deny the use of sugar, which is a luxury that may be safely enjoyed in moderation. A due proportion of salt should always accompany a vegetable diet.

***Beverage.*** When we turn to consider the proper beverage of childhood, we meet an abuse which calls for the severest reprehension. One might imagine that all persons endowed with common sense would understand that pure water, with or without a proportion of milk, or well made toast-and-water, must be the proper drink during the excitable period of childhood ; but unhappily we are compelled to advert to the fact, that some parents and nurses—not knowing the nature of what they do, have the pernicious habit of administering to children, not only tea and coffee, but wine, malt liquors, or even—generally as a cure for some slight complaint—ardent spirits ! It is a duty to explain clearly the tendency of this practice. It tends to injure the child and the future man, not only physically, but also morally. Proofs of this are unhappily too abundant : there can be no dispute on the point. Whatever may be said regarding the use or the abuse of wine, ale, or spirits, with regard to the *adult* constitution, it must be observed that on the delicate, excitable, and impressible constitution of children, such stimulants act with a tenfold pernicious effect, hurrying on the circulation —naturally quick in youth—vitiating the stomach, disturbing the nervous system, and producing an undue afflux of blood to the head. Common sense, without any help from physiology, might surely put down the practice against which we direct this paragraph. If wine or other alcoholic stimulants are suitable *medicines* to revive the languid circulation and sustain the animal warmth in cold and feeble old age, how can they be proper articles of diet for the precisely contrary condition of childhood? The fire required in the midst of December's snow is surely not wanted in the height of summer warmth! A spur to the youthful pulse is surely not required when it naturally runs at a pace which would indicate fever in an adult! Besides, the growth of the appetite for alcoholic stimulants, when frequently indulged, is well known to be rapid; and I would therefore ask, if a child of six years of age is allowed to drink a glass of port or sherry wine then, *what* and *how much* liquor may he be expected to take when he is a man of sixty? I would lay it down as a rule, that no alcoholic stimulants should be tasted during childhood. The few exceptions to this rule are cases which should be referred to medical treatment. If a medical man is consulted with regard to administering to a child a dose of rhubarb and magnesia, he should in all consistency be consulted before such a medicine as *port wine* is given.

*Artificial Feeding.* Hitherto, I have proceeded on the supposition that the infant has been nourished either by the mother or a well chosen nurse. But there may be cases in which it would be highly improper to allow the mother to suckle her own babe, while it may be difficult to employ the services of a good wet-nurse. In such a case, we must, with the utmost caution, employ a system of *artificial feeding.* On this point I must especially request parents to bestow the greatest care and attention; for, as the mode of artificial feeding is plainly a deviation from the plan of nature, we should study to make such a breach of rule as small as the circumstances of the case will allow. *A very considerable part of the mortality of infants reared by hand is the result of errors, respecting either the quality or the quantity of the artificial food administered.*

In the first place, I would observe that where an infant is to be brought up by hand, or where there exists a probability that ere long artificial feeding will have to be resorted to, it ought not to be put to the breast at all; weaning it after it has been accustomed to the breast-milk for a few weeks, is exposing it to imminent danger.

According to the principles already laid down, it is clear that the artificial food of infancy should form the *best possible imitation* of the natural milk which *ought* to be the diet of the infant. In order to succeed in this imitation, we must carefully study the properties of the original. Milk is the perfect form in which nature presents to us the three essential constituents, *saccharine*, *oily*, and *albuminous* matters, necessary to support infant life. It may be resolved into three organised compounds, which we may designate by the familiar terms, *cream*, *curd*, and *whey*. These three constituents vary in proportions in the milk of various animals, as the following table will show:—

| Properties. | Human. | Cow's milk. | Goat's milk. | Asses' milk. |
|---|---|---|---|---|
| Casein .......... | 2 95 | 4·48 | 4·02 | 1·82 |
| Butter .......... | 5·20 | 3·13 | 3·32 | 0·11 |
| Sugar .......... | 6·34 | 4·77 | 5·28 | 6·08 |
| Saline matters .. | 0·45 | 0·60 | 0·58 | 0·34 |
| Water .......... | 85·06 | 87·02 | 86·80 | 91·65 |
| Total ........ | 100·00 | 100·00 | 100·00 | 100·00 |

We see, in the above table, that the milk of the cow contains a considerably greater proportion of *casein* (or cheese-like matter) than human milk. Now it should be observed that this *casein* is the least digestible of the constituents of milk; and from this fact we learn the necessity of *diluting* cow's milk, so as to adapt it to the tender organisation of the child. The milk of woman contains a larger proportion of butter than that of the cow; and hence is inferred the propriety of adding a small quantity of cream to the diluted cow's milk, given in feeding by hand. But the addition of cream is not so important as the proper dilution of the milk. The foregoing table also shows us that it is proper to add to the diluted cow's milk a small quantity of loaf sugar, to increase its resemblance, in the saccharine quality, to the milk supplied for the child by nature. Moist sugar may be used occasionally, if the infant's bowels are confined; but let this be noticed—the free use of sugar is not to be recommended; it tends to cloy the stomach and weaken the digestion, thus producing acidity, sour eructations, and flatulence.

For the first four or five months, let a mixture of fresh cow's milk and pure boiling water, in equal proportions, with a small quantity of loaf sugar, be used. For the first fortnight the proportion of water may often be advantageously increased to two-thirds; but, as a general rule, the best mixture for a healthy, full grown infant, will be of equal parts of milk and water. At the expiration of the fourth or fifth month, pure, fresh, undiluted milk may be *gradually* substituted for the diluted mixture. If, at any time, sickness or disorder of the bowels occur, either diminish the quantity, or try it somewhat more diluted, until these symptoms subside. If, on the other hand, no disorder of the stomach or bowels is produced, and the infant is not satisfied with the above mixture, gradually diminish the quantity of water, or try the addition of *one teaspoonful of cream* to four ounces of the mixture of milk and water.

The above *recipe* for an imitation of the mother's milk may appear to some nurses as a very poor and thin sort of diet; and they may, consequently, think it advisable to deviate from it by now and then giving to the infant more substantial fare, in the shape of gruel, panada, or some other farinaceous preparation. Here the physician has to contend with a very obstinate and firmly rooted prejudice. The nurse imagines that, because a cup of gruel contains a considerable quantity of nutriment (even for an adult) it must, therefore, be suitable to strengthen the infant, and never considers that, if the infant's stomach is not prepared to digest and assimilate such food, the effect must be injurious. Frequent observation of the bad consequences of this prevailing error among nurses leads me to state here, emphatically, that an infant brought up by hand is much safer and has a much better chance of thriving with the said mixture of milk and water, than when a stronger food is used. Why? Because the former is well digested, while the latter is not; and all indigestible food produces irritation and oppression. It is quite certain that the mortality among artificially fed infants (which is confessedly *very great*) may be considerably diminished by careful attention to this point.

If gruel, panada, and similar food must be condemned, when they are inconsiderately administered to the infant, what must be said of the practice of giving to a toothless child such substances as solid bread, potatoes, or the flesh of animals—roast beef, for instance. One might think that common sense would sufficiently show the absurdity of such treatment. Nature has given to all creatures intended to consume flesh suitable teeth to tear and masticate it, and the adult human being is furnished with such teeth; but look at the mouth of the infant, and observe for what purpose it was intended. The tender and toothless gums, the soft, full and prominent lips, the whole formation, admirably adapted for receiving a bland, fluid diet by suction, but totally unfitted for mastication,—do not these signs clearly show the impropriety of giving to infants the diet of adults?

With regard to the *quantity* of the first artificial food proper in the early stage of infancy; let it be considered that the stomach is small and unaccustomed to its functions. Let the diet of the infant be gently given in small quantities. Let the first symptom of indifference be noticed as a sign that the appetite is satisfied for the present. As a general rule, we may state that six or eight tablespoonfuls will be enough to be given at one time. It is true that, when too much is given, nature (infinitely wiser than the "cramming nurse") provides a remedy for the evil by vomiting. But even this very emphatic pronunciation of "no more!" from the stomach, does not convince the nurse of her error. She only observes it, and says, "Ah, 'tis a good sign! the child is healthy." This again is an error. It is certainly well that the stomach can thus throw off the superfluous matter, but it would be far better to make the vomiting unnecessary.

It will be understood, from all that has been said of the propriety of imitating the mother's milk as closely as possible, that the milk and water should always be given neither hot nor cold, but *warm*. *The milk,* however, *should not be warmed over a fire* (for by boiling its nutritive quality is diminished), but only by the admixture of the proper quantity of water previously heated, so as to raise it to the temperature of milk from the breast, namely, from ninety to ninety-five degrees Fahrenheit; and in all cases in which care is needed, a thermometer should be employed in order to ensure the food being always given at the same temperature. Great care is necessary, especially in towns, in order to obtain genuine cow's milk. It should be procured, from time to time, *fresh from one healthy cow, and not mixed with the milk from any other cow.* The milk sold in large towns is commonly adulterated with chalk, starch, flour, and even still more objectionable ingredients, as well as diluted with water. If, however, arrangements cannot be made to procure pure fresh milk from one cow, due allowance must be made for the dilution it has already undergone—and a smaller quantity of water will be required from the first.

Human milk is alkaline, and, even when kept for a considerable time, shows but little tendency to become sour. The milk of animals, in perfect health, likewise invariably presents an alkaline reaction, and cows when at grass form no exception to this rule. Comparatively slight causes, however, exert a marked influence upon the milk of the cow in this respect; and if the animal be shut up and stall-fed, its milk almost constantly acquires a strongly acid property,* a fact which of itself is sufficient to account for the symptoms of gastric and intestinal disorder so often produced by it in the case of children brought up in large towns. Whenever, therefore, the attempt is made to rear an infant by hand, under circumstances which render it impossible to obtain the milk of cows which are at pasture, it is desirable that the milk should be daily tested, and that any acidity should be neutralised by the addition of lime water or of finely-levigated chalk, in quantity just sufficient to impart to it a slightly alkaline reaction. If the bowels are inclined to be constipated, carbonate of magnesia may be substituted for the chalk. The possibility of the occurrence of this acidity, and of the various adulterations referred to, shews the necessity, when an infant who is brought up by hand fails in health, for making a careful inquiry into the source of the milk with which it is fed; and for examining the fluid both chemically and under the microscope, before proceeding to prescribe remedies for ailments which may be caused entirely by the unwholesome nature of its food.

The rules which have been given for artificial feeding will generally be found to succeed. If they are faithfully carried out in all cases where the infant cannot be nursed by its mother, the mortality at this period of life will be very considerably diminished. Among many illustrations of their successful application in practice, may be mentioned the following instance: A mother had attempted to rear by hand nine children in succession, in consequence of a physical inability to nurse them herself; and each of them had died before it had reached the age of twelve months. It was obvious that an improper diet had been used. After the tenth confinement, the child was treated in accordance with the principles herein laid down, and is now living and healthy.

It must be admitted, however, that some *rare* cases occur, in which every attempt to rear a child, by the most judicious course of artificial feeding, will fail. Five cases of this kind have come under my notice. A state of stupor comes on, which gradually terminates fatally. The remedy, of course, is a healthy wet-nurse.†

---

* See the results of Dr. MAYER's observations on cows in Berlin and its neighbourhood, in a valuable paper on the artificial feeding of infants, in the Transactions of the Obstetric Society in Berlin, 8vo, Berlin, 1846; and Dr. WEST's Lectures on the Diseases of Infancy and Childhood.

† See *Medical Times*, October 11, 1851; or Half-Yearly Abstract of the Medical Sciences, vol. xiv.

(*To be continued.*)

